# Trend of various adiposity indices in women with and without history of gestational diabetes: a population-based cohort study

**DOI:** 10.1186/s12902-019-0348-5

**Published:** 2019-02-19

**Authors:** Samira Behboudi-Gandevani, Fahimeh Ramezani Tehrani, Maryam Rahmati, Mina Amiri, Fereidoun Azizi

**Affiliations:** 1 Reproductive Endocrinology Research Center, Research Institute for Endocrine Sciences, Shahid Beheshti University of Medical Sciences, Tehran, Iran; 2 Endocrine Research Center, Research Institute for Endocrine Sciences, Shahid Beheshti University of Medical Sciences, No 24, Parvane Street, Yaman Street, Velenjak, Tehran, Iran; 3 Department of Epidemiology and Biostatistics, School of Public Health, Tehran University of Medical Sciences, Tehran, Iran

**Keywords:** Adiposity indices, Gestational diabetes, Obesity, Trend, Tehran lipid and glucose study (TLGS)

## Abstract

**Background:**

Data of studies focusing on the trends of adiposity indices among women with prior gestational diabetes mellitus (GDM), are limited and controversial. The aim of this study was to compare overtime trends of adiposity indices in women with and without history of GDM, using data of the long term longitudinal population-based Tehran-Lipid and Glucose-Study (TLGS).

**Methods:**

A total of 3395 eligible women aged (20–50 years), including 801 women with prior history of GDM and 2594 non-GDM controls were recruited from among Tehran-Lipid and Glucose-Study participants. Generalized estimating equations were applied to assess the time trend of adiposity indices including obesity, central obesity, body mass index (BMI), lipid accumulation product index (LAP), visceral adiposity index (VAI) and a body shape index (ABSI) in women with prior GDM and the non-GDM groups after further adjustment for age and BMI.

**Results:**

Median follow-up years for the GDM and non-GDM groups were 12.12 and 11.62 years, respectively. Women with GDM at initiation had worse adiposity indices than their healthy counterparts. While overall odds of obesity in women with prior GDM were significantly higher those of the non-GDM groups (OR: 1.35; 95% CI, 1.03–1.7; *P* = 0.03), both these groups overtime revealed a positive trend in obesity (P _trend_ < 0.001), an incremental trend which was less pronounced in GDM women (OR: 0.87; 95% CI, 0.80, 0.95; P _interaction_ = 0.001). Women with prior GDM had higher odds of central obesity, compared to non-GDM groups (OR: 1.44; 95% CI, 1.06–1.96; *P* = 0.02) and showed a significant an incremental trend overtime for both groups (P _trend_ < 0.001 for both) without statistically significant interaction in terms of their GDM status (P _interaction_ = 0.134). Mean VAI in women with prior GDM was significantly higher than the non-GDM group (19.7, 95%CI: 6.24, 33.15, *P* = 0.004), although both groups overtime experienced a negative trend (− 10.9, 95%CI: -13.1, − 2.1, *P* < 0.001); the GDM group showed a higher decrease in VAI (mean changes: -6.62; 95% CI, − 11,-2.1; P _interaction_ = 0.001). However overtime there was a positive trend in LAP and ABSI among both women with and without prior-GDM, though the mean changes were less obvious in women with prior GDM.

**Conclusion:**

Women with prior GDM gained better control of their adiposity than non-GDM women. Nevertheless the increasing numbers of individuals with GDM and uncontrolled adiposity indices, require prompt attention be paid to the issue.

## Background

Obesity and gestational diabetes mellitus (GDM), two major epidemiologic concerns worldwide, in different age groups, ethnicities and socioeconomic statuses are increasing worldwide [1]. Obesity as a chronic low-grade inflammatory disease constitutes an important risk factor for morbidity and mortality [2], emphasizing the necessity of the early detection of risk factors and identification of at risk populations. Women with the history of GDM are one of these challenging high-risk groups that influence approximately 2–20% of all pregnancies [3]. It is well documented that maternal obesity is associated with GDM, and greater BMI increases the likelihood for insulin therapy later in life [4]. In a recent study of 22,351 women, each one unit increase of body mass index (BMI) in pregnancy, increased the odds of GDM in a subsequent pregnancy [5]. Obese women enter pregnancy with preexisting insulin resistance (IR) which worsens with advancing gestation [6, 7]. It has been shown that insulin-induced glucose transport in the human skeletal muscle fibers of obese women is suppressed in late pregnancy, and more so in those who develop GDM [8].

Since prior GDM is a well-known risk factor for overt type 2 diabetes [9], expert international societies recommend the promotion of healthy lifestyles that may affect adiposity indices in women with this status; increased fat mass is associated with elevated adipocytokine and proinflammatory cytokines, insulin resistance, increased risk of developing adverse outcomes of diabetes, hypertriglyceridemia, hypercholesterolemia, hypertension, atherosclerosis, and a higher mortality rate [10]. Several methods are used to assess the content and distribution of body fat. BMI assessment is commonly used for evaluation of obesity and prediction of adverse cardio-metabolic outcomes [11]. Recently some metabolic parameters of lipids were added to adiposity indices for better prognostic evaluation of cardio-metabolic adverse events [12]. However, less is known regarding the subsequent trend of obesity and other complex adiposity indexes, including lipid accumulation product (LAP), visceral adiposity index (VAI), a body shape index (ABSI) following delivery in women with a history of GDM, these women are at risk of lifelong complications, for which they need to be followed regularly to prevent any adverse events. Considering the lack of population-based studies and the lack of evidence about adiposity changes in women with history of gestational diabetes in comparison to their normal counterparts, this study aimed to compare the overtime trend of adiposity indexes in women with and without history of gestational diabetes mellitus (GDM), using data of the long term longitudinal population-based Tehran-Lipid and Glucose-Study (TLGS).

## Methods

The ethics committee of the Research Institute for Endocrine Sciences approved the study and written informed consent was obtained from all subjects before initiation of the study, which was conducted within the framework of the TLGS, an observational long term population-based, ongoing longitudinal study, initiated in 1998 to evaluate the prevalence and risk factors of non-communicable diseases in men and women [13]; eventually a total of 15,005 individuals, aged ≥3 years, were followed every 3 years follow-up, to document data on demographic, anthropometric, reproductive and metabolic characteristics, general physical examinations as well as laboratory measurements. Details of the examinations have previously been published elsewhere [13].

### Sample selection

A total of 4076 reproductive aged women, aged 20–50 years, with at least one previous term pregnancy at initiation of the study met the eligibility criteria; of these, 681 (16.7%) women were took part only at the one baseline visit (without any follow-up visits) were categorized “lost to follow-up”. Of the remaining 3395 women, 801 (23.6%) women with a history of prior GDM and 2594 (76.4%) without history of GDM, were followed at 3 year intervals. Of them, 737 (92%) of subjects with GDM, and 2283 (88%) in the non-GDM group had at least two follow-up visits. Also, 1894 (73%) and 657 (82%) of the subjects had at least three follow-up visits in non-GDM and GDM groups, respectively. The risk of obesity and central obesity and the trend of all adiposity indices were assessed overtime in both groups.

### Anthropometrics and biochemical measurements

Weight and height were measured, in the standing position with participants wearing minimal clothing, by trained staff using standardized procedures and calibrated equipment; WC was measured midway between the lower rib margin and the iliac-crest at the level of the umbilicus, at the end of a gentle expiration. Hip circumference (HC) was measured using an unstreched measuring tape to the nearest 0.1 cm.

Fasting blood samples were collected after 12 h of fasting (enzymatic colorimetric method, glucose oxidas kit, Pars Azmoon kit, Iran, inter and intra assay < 2.2%). Total cholesterol (TC) was measured (enzymatic colorimetric method with cholesterol esterase and cholesterol oxidase). HDL-C was assayed after precipitation of the apolipoprotein B (apo B)-containing lipoproteins with phosphotungistic acid. TG was assayed using glycerol phosphate oxidase. Intra- and inter-assay coefficients of variations for TC, HDL-C, and TG were below 1.9, 3, and 2.1%, respectively. Analyses were performed using related kits (Pars Azmon Inc., Tehran, Iran) and a Selecta 2 autoanalyzer (Vital Scientific, Spankeren, Netherlands). To calculate LDL-C, the modified Friedewald equation was used [14].

### Terms definitions

Since study initiation was in 1998, GDM screening and diagnosis were based on the World Health Organization (WHO) screening strategy [15]; hence, all pregnant women with a negative history of diabetes underwent the 75-g, 2 h oral glucose tolerance test (OGTT) between 24 and 28 weeks of gestation. At time of data collection, information was obtained a history of GDM, using a self-reporting questionnaire. Obesity was defined as BMI ≥ 30 kg/m^2^ and central obesity as WC ≥ 90 cm [16]. Adiposity indexes were calculated using the following formula: BMI: [weight (in kilograms) / square of height (in meters)]; lipid accumulation product (LAP): [(waist (cm)-58) * TG concentration (mmol/l)] [17]; female visceral adiposity index (VAI): [WC / 36.58 + (1.89* BMI) * (TG / 0.81) * (1.52/HDL-C)] (10); a body shape index (ABSI) = [WC (cm) / [BMI ^2/3^ * height (m)^1/2^] [18].

### Statistical analysis

Continuous variables were evaluated for normality using the one sample Kolmogorov-Smirnoff; skewed variables were log-transformed to normality before data analysis and are presented as mean or geographic mean (standard deviation), as appropriate. Categorical variables are expressed as percentages. Baseline characteristics were compared between the GDM and non-GDM groups using the student *t* test or chi-square test for continuous or categorical data, respectively. To investigate secular longitudinal trends of adiposity indices, including obesity, central obesity, BMI, LAP, VAI and ABSI, the generalized estimating equation (GEE) was used. Models for the examination of time trend were fitted separately for GDM and non-GDM groups and marginal (age-BMI-adjusted) means with *P* values for trends have been reported for each group. The interaction between the GDM status and each phase of the study was checked in a separate model; for this purpose, we entered the cross-product term (interaction term) in a single model including both GDM and non-GDM subjects and this analysis was performed on data of the first follow-up. All individuals were required to have data on at least one of the four follow-up visits. Predictors were: time (follow-up years), GDM status, and an interaction of these two (follow-up years × GDM group). This model was adjusted for age, BMI, and baseline status of each parameter. Statistical analysis was performed using the software package STATA (version 12; STATA Inc., College station, TX, USA); significance level was set at *p* < 0.05, and 95% confidence interval.

## Results

**Fig. 1:**
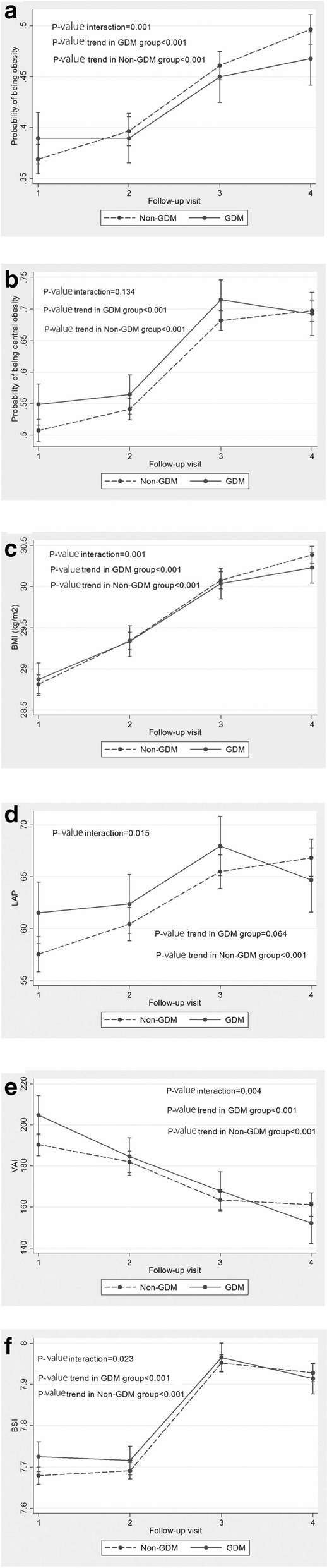
Generalized estimating equation (GEE) measures; Mean changes follow-ups between GDM and non-GDM groups assuming the interaction between time and the study group and also adjusting for age, BMI, and baseline parameters. A: obesity, B: central obesity, C: body mass index, D: lipid accumulation product, E: female visceral adiposity index, F: a body shape index

The median and interquartile range for follow-up years of GDM and non-GDM groups were: 12.12 (8.09–13.51) and 11.62 (6.26–13.14), respectively. Baseline characteristics of the subjects are presented in Table 1. Compared to non-GDM controls, women with GDM were more likely to be older, [38.1 (7.9) versus 35.3 (7.8) years (*P* < 0.001)], and had significantly higher WC [91.4 (11.5) vs. 86.04 (11.1) cm, *P* < 0.001], BMI [(29.2 (4.75) vs. 27.3 (4.5) kg/m^2^, *P* < 0.001] and WHR [(0.8 (0.07) vs. 0.8 (0.07), *P* < 0.001]. The prevalence of comorbidities of obesity and central obesity among women with history of GDM was significantly higher, compared to non-GDM groups (41.4% vs. 25.4%, *P* < 0.001) and (56.4% vs. 37%, *P* < 0.001), respectively. Moreover, at the beginning of the study, there were statistically significant differences between the two groups in terms of lipid profiles of the women, including total cholesterol, triglycerides, low-density lipoprotein cholesterol (LDL-C) and high-density lipoprotein cholesterol (HDL-C) (Table 1). Figure 1 illustrates age-BMI adjusted trends of adiposity indices in the GDM and non-GDM groups. (Fig. 1a-f) While the overall odds of obesity in women with prior GDM were significantly higher than the non-GDM group (OR: 1.35; 95% CI, 1.03–1.7; *P* = 0.03); both groups demonstrated a positive overtime trend in obesity (P _trend_ < 0.001), although this incremental trend was less pronounced in GDM women (OR: 0.87; 95% CI, 0.80, 0.95; P _interaction_ = 0.001) (Table 2). Figure 1 and Table 2 demonstrate approximately the same findings following comparison of changes in BMI trends between GDM and the non-GDM groups. Meanwhile, women with prior GDM had higher odds of central obesity, compared to their non-GDM peers (OR: 1.44; 95% CI, 1.06–1.96; *P* = 0.02) (Table 2); central obesity showed a significant incremental trend over time for both GDM and non-GDM groups (P _trend_ < 0.001 for both), without any statistically significant interaction in terms of their GDM status (P _interaction_ = 0.134). Mean LAP in women with prior GDM was significantly higher than those in the non-GDM group (5.96, 95%CI: 1.68, 10.23, *P* = 0.006). Although women with GDM experienced a non-significant increase (P _trend_ = 0.064), their non-GDM peers generally showed a statistically significant increase in their LAP index (P _trend_ = 0.001); mean changes was less noticeable in women with prior GDM (mean changes: -0.02; 95% CI, − 0.04,-0.003; P _interaction_ = 0.023). However, mean VAI in women with prior GDM was significantly higher than in the non-GDM group (19.7, 95%CI: 6.24, 33.15, *P* = 0.004); however, both groups overtime showed a negative trend (− 10.9 at each visit, 95%CI: -13.1, − 2.1, *P* < 0.001), with the GDM group however showing more decrease (mean changes: -6.62; 95% CI, − 11,-2.1; P _interaction_ = 0.001). The overall mean of ABSI in GDM women was 0.07 points higher than the non-GDM group (95%CI: 0.02–0.12, *P* = 0.008). Although overtime a positive trend was seen in the ABSI in both groups (0.10 per visit, 95%CI: 0.09–0.11, *P* < 0.001), this increasing trend was less pronounced in GDM women (mean changes: -0.02; 95% CI, − 0.04,-0.003; P _interaction_ = 0.023).

**Table 1:** Baseline characteristics of study participants according to their history of gestational diabetes mellitus (GDM)

Variables	GDM (*N* = 801)	Non-GDM (*N* = 2594)	*P*- value
Age (years)	38.10 (7.93)	35.31 (7.88)	0.001
BMI (kg/m^2^)	29.23 (4.75)	27.34 (4.54)	0.001
WC (cm)	91.40 (11.75)	86.04 (11.17)	0.001
WHR	0.85 (0.07)	0.82 (0.07)	0.001
WHtR	0.58 (0.8)	0.55 (0.7)	0.001
Obesity, n (%)	319 (41.4%)	627 (25.4%)	0.001
Central obesity, n (%)	429 (56.4%)	905 (37%)	0.001
TG (mmol/L) ^a^	0.51 (0.55)	0.33 (0.53)	0.001
TC (mmol/L)	5.35 (1.10)	5.14 (1.10)	0.001
LDL-C (mmol/L)	3.40 (0.89)	3.24 (0.91)	0.001
HDL-C (mmol/L)	1.12 (0.28)	1.15 (0.29)	0.001

*BMI* body mass index, *WC* waist circumference, *WHR* waist to hip ratio, *WHtR* waist t height ratio, *TG* triglyceride, *TC* total cholesterol, *LDL-C* low-density lipoprotein cholesterol, *HDL-C* high-density lipoprotein

^a^LN, transformed

**Table 2:** Estimation of the generalized estimating equation (GEE) model in women with and without gestational diabetes mellitus (GDM)

Variables	Coefficient^a^	95% Wald Confidence Interval	*P*-Value
Obesity			
GDM	1.35	(1.03, 1.76)	0.03
Non-GDM	Reference	–	–
Age (years)	0.99	(0.98, 1.01)	0.78
BMI (kg/m2)	1.82	(1.73, 1.91)	< 0.001
Baseline obesity	1.85	(1.40, 2.45)	< 0.001
Time (years)	1.43	(1.37, 1.49)	< 0.001
GDM*Time	0.87	(0.80, 0.95)	0.001
Non-GDM*Time	Reference	–	–
Central obesity			
GDM	1.44	(1.06, 1.96)	0.02
Non-GDM	Reference	–	–
Age(years)	1.04	(1.03, 1.05)	< 0.001
BMI(kg/m2)	1.36	(1.33, 1.39)	< 0.001
Baseline central obesity	2.70	(2.27, 3.21)	< 0.001
Time (years)	1.62	(1.53, 1.71)	< 0.001
GDM*Time	0.92	(0.82, 1.03)	0.13
Non-GDM*Time	Reference	–	–
BMI			
GDM	0.19	(−0.02, 0.41)	0.08
Non-GDM	Reference	–	–
Age(years)	-0.02	(−0.03, − 0.01)	0.001
BMI (kg/m2)	0.91	(0.89, 0.92)	< 0.001
Time (years)	0.42	(0.39, 0.45)	< 0.001
GDM*Time	−0.10	(− 0.15, − 0.04)	0.001
Non-GDM*Time	Reference	–	–
LAP			
GDM	5.96	(1.68, 10.23)	0.006
Non-GDM	Reference	–	–
Age(years)	.39	(.25, .53)	< 0.001
BMI (kg/m2)	1.03	(.76, 1.31)	< 0.001
Baseline LAP	.61	(.58, .64)	< 0.001
Time (years)	3.32	(2.61, 4.03)	< 0.001
GDM*Time	−1.77	(−3.19, −.35)	0.015
Non-GDM*Time	Reference	–	–
VAI			
GDM	19.70	(6.24, 33.15)	0.004
Non-GDM	Reference	–	–
Age(years)	.75	(.31, 1.18)	0.001
BMI (kg/m2)	2.02	(1.22, 2.81)	< 0.001
Baseline VAI	.55	(.52, .57)	< 0.001
Time (years)	−10.94	(−13.18, −8.70)	< 0.001
GDM*Time	−6.62	(−11.07, −2.16)	0.004
Non-GDM*Time	Reference	–	–
ABSI			
GDM	.07	(.02, .12)	0.008
Non-GDM	Reference	–	–
Age(years)	.02	(.01, .02)	< 0.001
BMI (kg/m2)	−.004	(−.01, −.002)	0.002
Baseline BSI	.34	(.31, .36)	< 0.001
Time (years)	.10	(.09, .11)	< 0.001
GDM*Time	−.02	(−.04, −.003)	0.023
Non-GDM*Time	Reference	–	–

^a^ Odds Ratio for obesity and central obesity and means for other variables

## Discussion

Our large population-based cohort study, over a decade long follow-up demonstrated that despite overall higher obesity, central obesity and adiposity indices in women with prior GDM compared to their non-GDM counterparts, these indices remained more stable in the former compared to latter who experienced worse changes. Despite higher BMI and worse adiposity indices in women with a history of GDM, compared to non-GDM ones, these gaps converge gradually; as seen in controls who bridged the gap in previous differences of risk profiles over time.

A wealth of literature supports the association between GDM and obesity [19, 20], For instance, Ramos et al. in a retrospective study of 22,658 women reported that the risk of GDM in obese women was significantly (2-fold) higher than in their non-obese peers [21]. However, limited studies have examined the trend of obesity and adiposity changes in women with and without GDM over time. In this respect, Bennett et al. in the Coronary Artery Risk Development in Young Adults (CARDIA) study compared pre- to post-pregnancy changes in weight, BMI, WC in women with (*n* = 137) and without GDM (*n* = 1637) during 20 years of follow-up; they showed that women with GDM had higher pre-pregnancy weight and BMI, compared to non-GDM ones. While following pregnancy, BMI and weight in both groups of women with and without GDM increased, pre- to post- pregnancy changes in body weight, BMI and waist circumference did not differ between these two groups [22].

Indicators of adiposity may be markers for changes in energy metabolism that influence diabetes and cardiovascular disease progression, and mortality. BMI, a simple indicator of obesity is the most commonly used approach to characterize obesity in individuals, it however has some important limitations including a lack of ability to differentiate between fat and muscle mass and not affirming fat distribution [23]. Among other more accurate measures, the combined indices of VAI and LAP have been proposed as valuable indicators of visceral adipose function [17, 24]. VAI is gender-specific indicator based on WC, BMI, TGs, and HDL and LAP is based on TGs and WC; both are known to reliably predict insulin resistance and diabetes mellitus, metabolic syndrome, cardiovascular events as well as all-cause mortality in non-diabetic patients [25–27]. These adiposity indicators include both anthropometric and metabolic parameters that may indirectly reflect other non-classical risk factors in the development of adverse cardio-metabolic events such as altered secretion of adipokines, elevated lipolysis and plasma free fatty acids, which are not specified by anthropometric or metabolic indicators separately [10].

Our findings showed that while LAP and ABSI had an increasing trend over time, the trend of VAI significantly decreased in both GDM and non-GDM groups, results which may highly correlate with the increasing trend of TG, BMI and WC overtime reported by other studies [28–30]. In addition, it was shown that WC adjusted for BMI was increased to a larger extent amongst younger versus older women [30]. Moreover, based on a formula, both LAP and ABSI have been directly related to WC and also have an inverse association with BMI; it is assumed that the effect of BMI is greater in VAI than LAP, a finding that supports previous evidence demonstrating that the cut-off points of VAI identifying visceral adipose dysfunction decrease with age [31]. Our study indicated that women with prior GDM had significantly higher BMI at initiation of study, although higher BMI in women with GDM is decreases overtime most probably due to the considerable weight loss in GDM group and weight gain among the non-GDM women; this may be related to the difference in life style of the subjects. However, due to certain underlying disturbances in GDM, which may be further exacerbated by fat accretion during pregnancy, these women have an increased risk of obesity, diabetes and other cardio-metabolic diseases [9]. In a long term follow up study, Minooee et al. reported that the incidence rate of diagnosed diabetes among women with prior GDM was significantly higher than in their non-GDM counterparts (9/1000 for women with GDM and 4/1000 for non-GDM ones). Moreover, women with prior GDM had shorter median time for developing diabetes compared to those without GDM [6.95 years (IQ: 4.22–10.71)] vs, [8.45 years [(IQ: 5.08–10.89)] [32]. Hence the aforementioned studies and professional societies strongly recommend screening, monitoring and promotion of lifestyle changes to decrease the heavy burden of these adverse outcomes in the future [28, 33–35]. Increased sensitivity of health care providers for diabetes surveillance and prevention in women with prior GDM, may improve the awareness of these women regarding implementation of lifestyle changes to prevent diabetes. We assume that women with prior GDM, as highly selected individuals, are more likely to modify their lifestyle with various interventions including diet, exercise and metformin therapy. The prevalence of obesity, sedentary lifestyle, and nutritional transition have been growing among the Iranian population in the past decade; therefore, the risk of impaired adiposity indices in women with prior GDM in later life may be lower than initially anticipated. In this respect, Ratner et al. showed that metformin and lifestyle interventions in women with the history of GDM could steadily decrease weight gain as well as reduce the future risk of obesity and overt diabetes [35]. However, all women, regardless of GDM diagnosis experienced an increase in abdominal and visceral adiposity overtime, following pregnancy, which could be associated with a greater risk of adverse cardio-metabolic outcomes. Our results additionally highlight the necessity to encourage all women to make healthy lifestyle modifications following after pregnancy.

The main strengths of our study is its methodology as a long term prospective community-based study with long follow-up. Low levels of lost to follow-up and precise measurements of anthropometric and metabolic parameters are other strengths of this study.

However, this study is limited by investigating only an Iranian population and cannot be extrapolated to other population, which emphasizes the need for more cohort studies in other ethnic populations. Furthermore, GDM diagnosis was self-reported, which may be limited by recall bias, although, the universal screening strategy of GDM in Iran and subsequent monitoring and treatment of GDM may restrict this bias. Furthermore, we did not collect data regarding some lifestyle modifications and dietary habits; the healthy cohort effect (knowledge about GDM and paying more attention to themselves) may have influenced their lifestyle habits, causing desirable adiposity status in participants; which led to our optimistic findings.

## Conclusion

To the best of our knowledge this is one of the pioneer studies with a long term follow-up to evaluate the impact of GDM on trends of obesity indices. Its results demonstrate that despite higher rates of obesity and adiposity indices in women with prior GDM, compared to their non-GDM counterparts, these additional risks decreases gradually; women with prior GDM gained better control of their adiposity than non-GDM women. Nevertheless the increasing numbers of people with GDM and uncontrolled adiposity indices, require prompt attention be paid to the issue.

## Abbreviations

ABSI: A body shape index
BMI: Body mass index
GDM: Gestational diabetes mellitus
GEE: Generalized estimating equation
HC: Hip circumference
HDL-C: High-density lipoprotein cholesterol
LAP: Lipid accumulation product
LDL-C: Low-density lipoprotein cholesterol
OGTT: Oral glucose tolerance test
TC: Total cholesterol
TG: Triglyceride
TLGS: Tehran lipid and glucose study
VAI: Visceral adiposity index
WC: Waist circumference
WHO: World Health Organization
